# Impact of Imipenem on Cognitive Function, Anxiety-like Behaviour, Neurogenesis, and Astrogliosis in Pentylenetetrazol-Kindled Rats

**DOI:** 10.3390/antibiotics15080728

**Published:** 2026-07-27

**Authors:** Leonardo Araújo-Andrade, Pedro Nogueira, Bárbara Caetano-Mota, Nuno Lima, Ana Silva, Pedro A. Pereira, M. D. Madeira, Armando Cardoso

**Affiliations:** 1 Unit of Anatomy, Department of Biomedicine, Faculty of Medicine, University of Porto, Alameda Professor Hernâni Monteiro, 4200-319 Porto, Portugal; 2 NeuroGen Research Group, Center for Health Technology and Services Research (CINTESIS), Rua Dr. Plácido da Costa, 4200-450 Porto, Portugal; 3 RISE-Health, Unit of Anatomy, Department of Biomedicine, Faculty of Medicine, University of Porto, Alameda Professor Hernâni Monteiro, 4200-319 Porto, Portugal; 4 Unidade Local de Saúde de São João–Centro Hospitalar Universitário de São João, Alameda Professor Hernâni Monteiro, 4200-319 Porto, Portugal

**Keywords:** imipenem, hippocampus, epilepsy, pentylenetetrazol, learning, memory, anxiety, neurogenesis, astrogliosis

## Abstract

**Background/Objectives:** Adverse neurological events caused by imipenem are usually more frequent in patients with epilepsy. This study investigates the effects of imipenem on hippocampal neurogenesis, astroglial activation, and behaviour in animals with pro-epileptogenic alterations. **Methods**: Five-month-old Wistar rats were divided into four groups: control (CT), pentylenetetrazol (PTZ), imipenem-treated group (IM), and imipenem after PTZ-kindling (IM-PTZ). Epileptogenesis was induced by PTZ-kindling for 28 days, followed by 10 days of imipenem (40 mg/kg) or saline injections. Rats underwent memory and anxiety tests followed by immunohistochemical analyses of doublecortin (DCX) and glial fibrillary acidic protein (GFAP) in the hippocampus. **Results:** Both kindled groups displayed comparable impairments in spatial learning and working memory, while imipenem alone did not alter behaviour. The IM-PTZ group failed to reach control-level performance only in the final Morris Water Maze acquisition trials, suggesting a deficit in memory consolidation rather than a total loss of retention, as the probe trial remained comparable across all groups. Although PTZ increased anxiety-like behaviour, no locomotor differences were observed in open-field test, confirming cognitive impairments were not due to motor deficits. These behavioural changes mirrored distinct cellular alterations: while PTZ alone triggered a compensatory increase in DCX neuronal density and astrocytic branching, imipenem co-administration suppressed this neurogenic response, bringing DCX to baseline. Furthermore, combined treatment induced an uncoupling of astrocytic response, marked by increased astrocyte density and reduced process complexity in dentate hilus and Cornus Ammonis 3 (CA3). **Conclusions:** These results suggest that imipenem modulates the cellular substrate of the pre-sensitized brain, interfering with hippocampal plasticity and glial compensatory mechanisms, rather than directly driving further behavioural deterioration.

## 1. Introduction

Carbapenems are broad-spectrum antibiotics reserved for infections caused by multi-drug-resistant bacteria and severe clinical cases when a narrower spectrum may leave out the causative agent [1]. Their spectrum is active against both Gram-negative and Gram-positive bacteria, and their pharmacodynamic and pharmacokinetic characteristics make them useful in various infectious foci [2]. Imipenem was the first carbapenem discovered and used in clinical practice [3]. It demonstrates superior efficacy against Gram-positive cocci compared to meropenem and, unlike ertapenem, maintains activity against *Pseudomonas aeruginosa* [4]. Imipenem is also more effective than other carbapenems against *Nocardia* spp., without the adverse effects of trimethoprim/sulfamethoxazole, making it a preferred choice for severe central nervous system (CNS) infections and initial intensive treatment against this bacterium [5].

In spite of the various positive properties of imipenem and its successful use in the treatment of various infections, adverse neurological effects, though rare, have been reported [6]. Although not fully elucidated, neurotoxicity due to carbapenems and other β-lactams is attributed to interference with the γ-Aminobutyric acid (GABA) system [7,8]. Interference with the GABA receptors in the same binding site as benzodiazepines reduces the expected inhibition effect and seizure threshold, potentiating the occurrence of pro-epileptic phenomena [9]. Animal studies have shown that the affinity of imipenem for GABA receptors is higher than that of other carbapenems and causes proconvulsive phenomena more easily than other carbapenems [10].

Neurological adverse effects of imipenem vary, ranging from severe convulsions to delirium and psychosis [6]. The likelihood of convulsions has been correlated with previously known epilepsy and increased serum concentrations of the antibiotic [11,12]. Retrospective studies corroborate results from animal studies, indicating a higher risk of convulsions in patients treated with imipenem compared to meropenem [13]. Central to this neurotoxic susceptibility is the hippocampal formation, a region with a high density of GABAergic receptors and extreme sensitivity to excitatory imbalances [14]. Specifically, the subfields of the dentate gyrus, CA3, Cornus Ammonis 1 (CA1) and subiculum play integrated roles in memory encoding and emotional regulation [15]. These subfields are also particularly vulnerable to the proconvulsive effects of β-lactams [16,17], which can lead to localized neuroinflammation and impaired neuroplasticity. Clinical studies also show, however, that even though patients treated with imipenem have a higher risk of convulsions, the association might be more complex than previously thought [18,19]. When using doses adjusted for renal function and body weight, the likelihood of convulsions appears to be independent of duration of imipenem therapy [18]. Therefore, pharmacodynamics and pharmacokinetics of imipenem, as well as its interference in the neuronal network, seem to play a key role in the occurrence of side effects.

Further research is required to address the knowledge gap surrounding imipenem’s adverse neurological mechanisms and impact, especially when pro-epileptogenic lesions are present. Previous animal studies on imipenem have frequently employed doses that are not representative of those used in clinical practice, limiting the translational relevance of their findings [10]. In addition, relatively few studies have investigated behavioural and cognitive effects of imipenem, and even fewer have addressed these outcomes in epileptic animal models [20]. Notably, in studies of epilepsy and convulsions, imipenem has typically been administered at supratherapeutic doses, reducing its clinical translation [20].

Given the need for further investigation into the neurobiological effects of relevant doses of imipenem, with translational potential, on the CNS specifically in the presence of pro-epileptogenic alterations, this study focused exclusively on the 40 mg/kg dose, previously associated with hippocampal and cerebellar impairment in juvenile Wistar rats [20]. Moreover, similar dosing has been used in experimental rat infection models to achieve therapeutically relevant pharmacodynamic exposure [21]. As the pharmacokinetic and pharmacodynamic profile of imipenem has been extensively characterized in both healthy and infected rats, dose selection in preclinical studies is guided by established pharmacodynamic targets, particularly the time that free drug concentrations remain above the minimum inhibitory concentration, rather than by direct mg/kg equivalence with human dosing [22,23,24]. Because the objective of the present study was to investigate the CNS effects of imipenem rather than to characterize its pharmacokinetics, the 40 mg/kg dose was selected based on this well-established preclinical evidence and its previously demonstrated neurobiological effects.

To induce epilepsy, we chose the pentylenetetrazol (PTZ)-induced kindling model, as this model is widely used to investigate epilepsy and epileptogenesis in rats, particularly when impaired GABAergic dysfunction is implicated [25,26]. PTZ is a competitive GABA receptor antagonist widely used to induce experimental epilepsy [25,26]. Whereas high doses elicit acute generalized seizures, repeated administration of subconvulsive doses leads to the progressive development of epilepsy through the process of chemical kindling [25,26]. PTZ antagonism causes paroxysmal depolarizing shifts in neurons by blocking the chloride channel of the GABA receptors, which suppresses inhibitory postsynaptic potentials and shifts the net balance of the neural network toward uncontrolled glutamate-mediated excitation [27]. Repeated administrations of PTZ, instead of causing an isolated severe seizure, as seen with a single high-dose administration, lead to chronic maladaptive neuroplasticity and structural modifications of the neuronal network [26]. Said alterations mimic the ones observed in patients with epilepsy; hence, the PTZ-kindling model is widely used to study epilepsy, as well as pro- and anti-epileptic drugs [28].

By employing a PTZ-kindling model and the aforementioned dose of imipenem, we aimed to determine whether imipenem exacerbates behavioural deficits and modulates brain plasticity under chronic preexisting pro-epileptogenic conditions. To this end, we evaluated spatial learning and memory, anxiety-like profiles, and locomotor activity, while also investigating underlying cellular changes in dentate gyrus, CA3, CA1 and subiculum subfields, through doublecortin (DCX) expression, as a marker of neurogenesis, and glial fibrillary acidic protein (GFAP)-immunoreactivity to assess astrogliosis and neuroinflammation.

## 2. Results

### 2.1. Morris Water Maze (MWM)

The MWM is a standard behavioural test in neuroscience used to examine spatial learning and memory in rodents. To investigate whether PTZ and imipenem alone or in combination influence these cognitive domains, two different MWM assessments were performed: reference memory and working memory.

#### 2.1.1. MWM—Reference Memory

**Figure 1:**
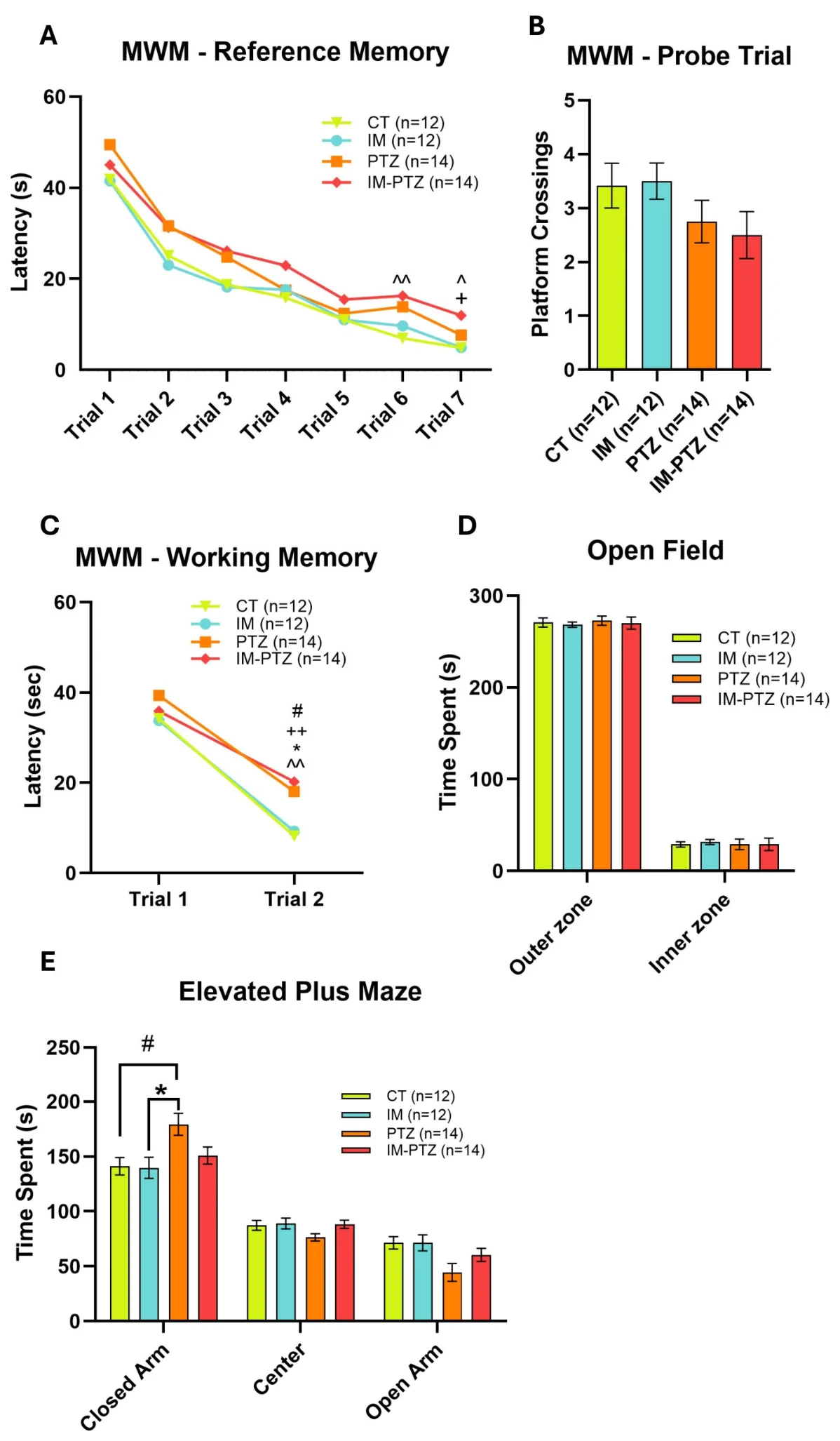
Results from memory and anxiety assessments. (**A**) Latency time (s) in the Morris water maze (MWM) during reference memory acquisition across trial blocks, expressed as mean ± SEM. The imipenem–pentylenetetrazol (IM–PTZ) group showed worse performance than controls (CT) in trial blocks 6 (*p* < 0.05), and 7 (*p* < 0.01), and worse than the imipenem (IM) group in trial block 7 (*p* < 0.01). (**B**) Analysis of the reference memory probe trial revealed no significant group differences in the mean number of crossings over the target platform location (mean ± SEM). (**C**) Time spent to reach the platform during the information (trial 1) and retention (trial 2) trials (mean ± SEM). Rats in the PTZ group performed worse than both the CT and IM groups (*p* < 0.05). Epileptic rats in the IM–PTZ group also performed worse than CT (*p* < 0.01) and IM (*p* < 0.05) groups but did not differ significantly from the PTZ group. (**D**) Time spent in the outer and inner zones of the open field test (mean ± SEM). All groups spent the majority of time in the outer zone, with no significant differences between groups. (**E**) Time spent in the closed arms, centre area, and open arms of the elevated plus maze (mean ± SEM). Rats in the PTZ group spent significantly more time in the closed arms compared to the CT and IM groups (*p* < 0.05). ^^^ *p* < 0.05, ^^^^ *p* < 0.01 IM-PTZ vs. CT; ^++^ *p* < 0.01 IM-PTZ vs. IM; ^#^ *p* < 0.05 PTZ vs. CT; * *p* < 0.05 PTZ vs. IM.

Long-term spatial learning was evaluated using the reference memory version of the MWM. As shown in Figure 1A, the average time (seconds) required to locate the hidden platform was recorded. Repeated-measures ANOVA indicated that all experimental groups demonstrated a progressive reduction in escape latency across the acquisition phase, reflecting improved learning of the platform location (F(6, 288) = 122.69, *p* < 0.0001). No significant main effect of drug administration (F(3, 48) = 2.674, *p* = 0.0577) or treatment x quadrant interaction (F(18, 288) = 0.523, *p* = 0.946) was found on the time required to locate the platform. Despite the lack of a global interaction, to further characterize the nature of the cognitive impairment, we focused our analysis on the terminal acquisition phase (trial blocks 6 and 7), where spatial learning performance reaches a plateau and task demand is maximal. A one-way ANOVA performed on trial blocks 6 and 7 revealed a significant effect of treatment on both trial blocks 6 (F(3, 48) = 3.241, *p* < 0.05) and 7 (F(3, 48) = 6.625, *p* < 0.01). Post hoc analysis using the Tukey HSD test confirmed that the IM-PTZ group exhibited significantly higher escape latencies compared to the control group (*p* < 0.05) in trial block 6. Consistent findings were observed for trial block 7, where the IM-PTZ group also showed significantly impaired performance compared to controls (*p* < 0.01) and IM (*p* < 0.01) groups.

#### 2.1.2. MWM—Reference Memory: Probe Test

As illustrated in Figure 1B, analysis of the probe trial data revealed no significant differences in the number of platform crossings among the treatment groups. This was confirmed by one-way ANOVA, which showed no significant effect of drug administration (F(3, 44) = 1.543, *p* = 0.2167).

#### 2.1.3. MWM—Working Memory

Working memory variation in the MWM test was used to assess short-term spatial memory. Figure 1C shows the mean latency, in seconds, needed to find the platform in the information (trial 1) and retention (trial 2) trials. Repeated-measures ANOVA revealed that there was a significant effect of drug administration (F(3, 48) = 6.039, *p* < 0.01) in the trial-block effect (F(1, 48) = 315.9, *p* < 0.0001), and of the interaction (F(3, 48) = 3.563, *p* < 0.05). Post hoc analysis showed that the PTZ animals spent more time finding the hidden platform in the retention trial compared to CT (*p* < 0.05) and IM animals (*p* < 0.05). In addition, IM-PTZ animals spent more time finding the platform than CT (*p* < 0.01) and IM animals (*p* < 0.05).

### 2.2. Open-Field Test

The open-field test assesses various aspects of a rodent’s behaviour, such as general activity and exploration habits. For anxiety-like behaviour, as shown in Figure 1D, the results showed that the time spent was significantly influenced by zone (F(1, 48) = 2263, *p* < 0.0001), but not by drug administration (F(3, 48) = 0.3020, *p* = 0.8238), with no drug administration x zone interaction (F(3, 48) = 0.8134, *p* = 0.9699). Rats from all groups spent more time in the outer zone than in the inner zone (*p* < 0.0001), with no differences between groups in either zone.

### 2.3. Elevated Plus Maze—EPM

The EPM test (Figure 1E) was used to assess anxiety-like behaviour in rodents. The results show that there was a significant effect of zone (F(2, 144) = 184.67, *p* < 0.0001) and drug administration x zone interaction (F(6, 144) = 5.713, *p* < 0.0001), but no main effect of drug administration (F(3, 144) = 0.0076, *p* = 0.999). This non-significant main effect, despite a significant interaction, indicates that drug-related differences were zone-dependent rather than globally affecting anxiety-like behaviour. Post hoc comparisons revealed that animals in the PTZ group spent significantly more time in the closed arms when compared to CT (*p* < 0.05) and IM animals (*p* < 0.05), while no significant differences were observed in time spent in the open arms or centre.

### 2.4. Neurogenesis

**Figure 2:**
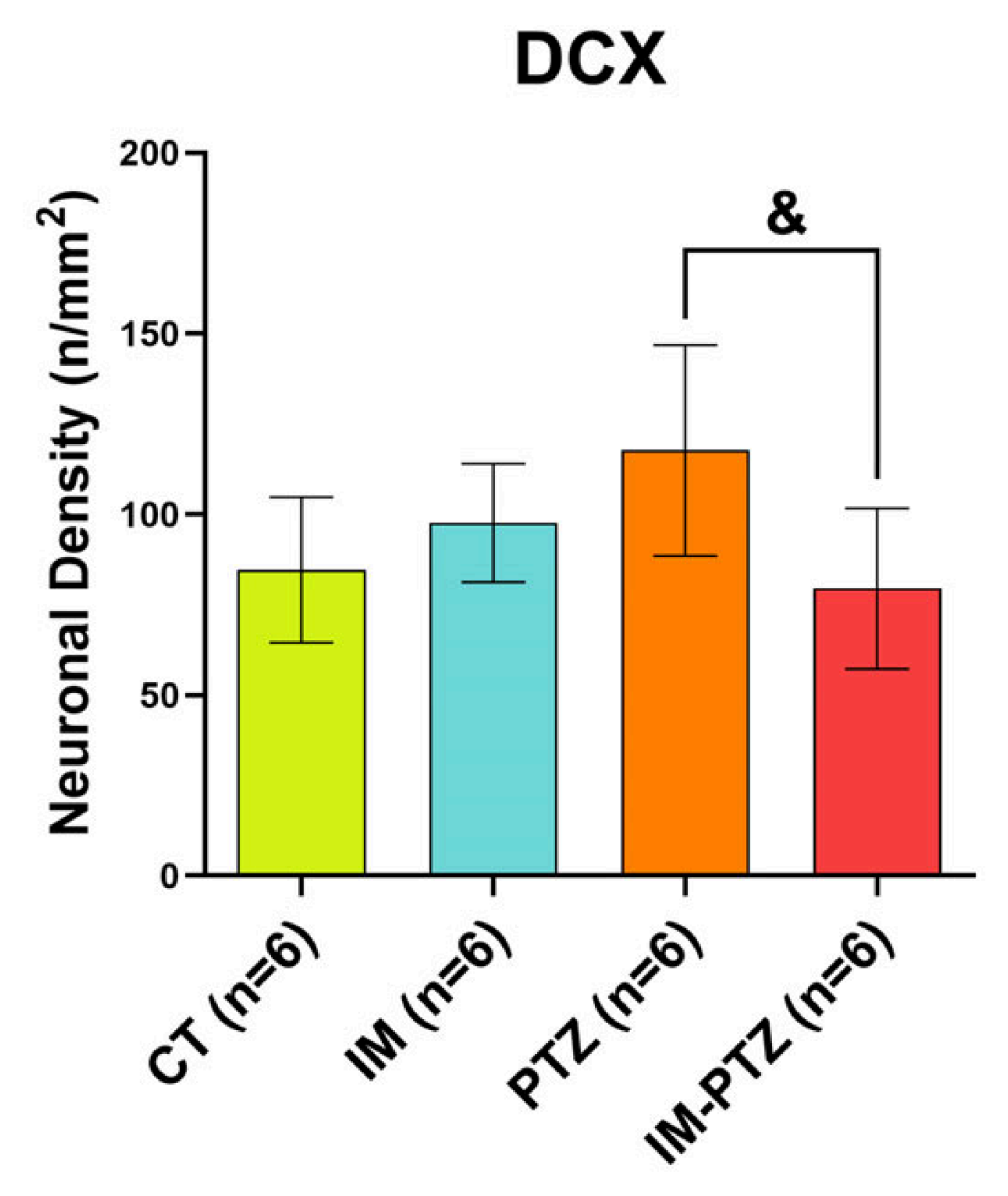
Effects of imipenem and PTZ on hippocampal DCX-IR neuronal density. —Histogram shows neuronal density of doublecortin-immunoreactive (DCX-IR) neurons in the subgranular layer of the hippocampus (mean ± SD). Pentylenetetrazol (PTZ) administration led to a tendency towards increase in DCX-IR density compared to control (CT) and imipenem (IM) groups that was not significant; PTZ rats treated with imipenem (IM-PTZ) showed a significant decrease in neuronal density when compared with the PTZ group (*p* < 0.05). ^&^ *p* < 0.05 IM-PTZ vs. PTZ.

**Figure 3:**
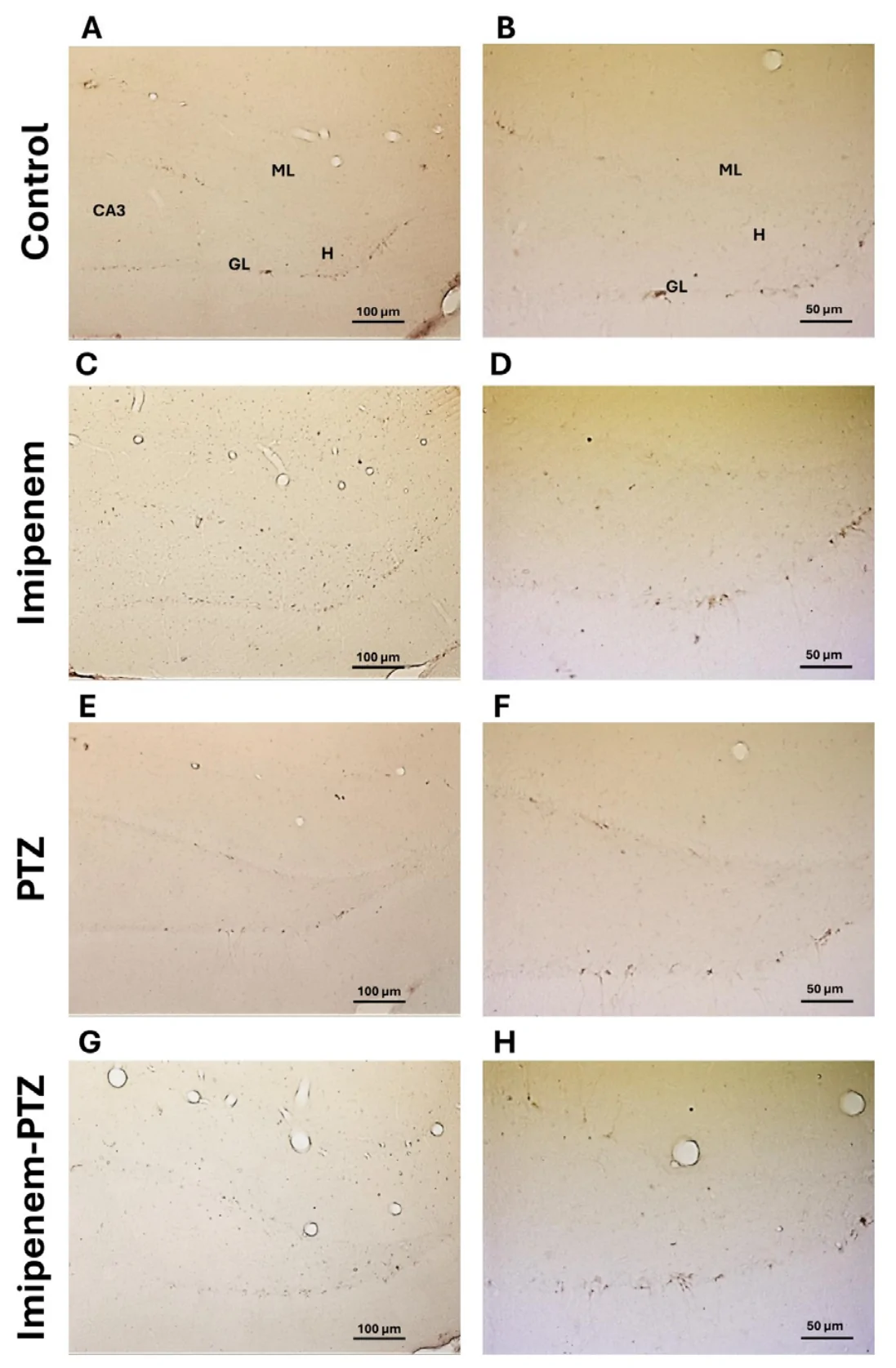
Effects of imipenem and PTZ on hippocampal DCX-immunoreactive neuronal density. Representative photomicrographs of the hippocampal formation from the experimental groups at different magnifications. Labels are included only in the first photomicrographs for reference and are omitted from the remaining images for clarity. (**A**,**B**) CT, (**C**,**D**) IM, (**E**,**F**) PTZ, and (**G**,**H**) IM–PTZ. ML, molecular layer of the dentate gyrus; GL, granular layer; H, dentate hilus; CA3, pyramidal layer of the CA3 hippocampal field.

Estimations of the areal density of DCX-immunoreactive (DCX-IR) neurons in the subgranular layer of the hippocampal formation are shown in Figure 2 and representative microphotographs from the different groups in Figure 3. The results showed a significant effect of drug administration on the number of DCX-IR neurons in the subgranular layer of the dentate gyrus (F(3, 20) = 3.460, *p* < 0.05). Although not reaching statistical significance, PTZ administration led to a visible trend of increased DCX-IR density compared to the control and imipenem groups. Post hoc analysis showed that IM-PTZ rats had a significant decrease in the neuronal density of DCX-IR neurons compared to PTZ rats (*p* < 0.05).

### 2.5. Astrogliosis

**Figure 4:**
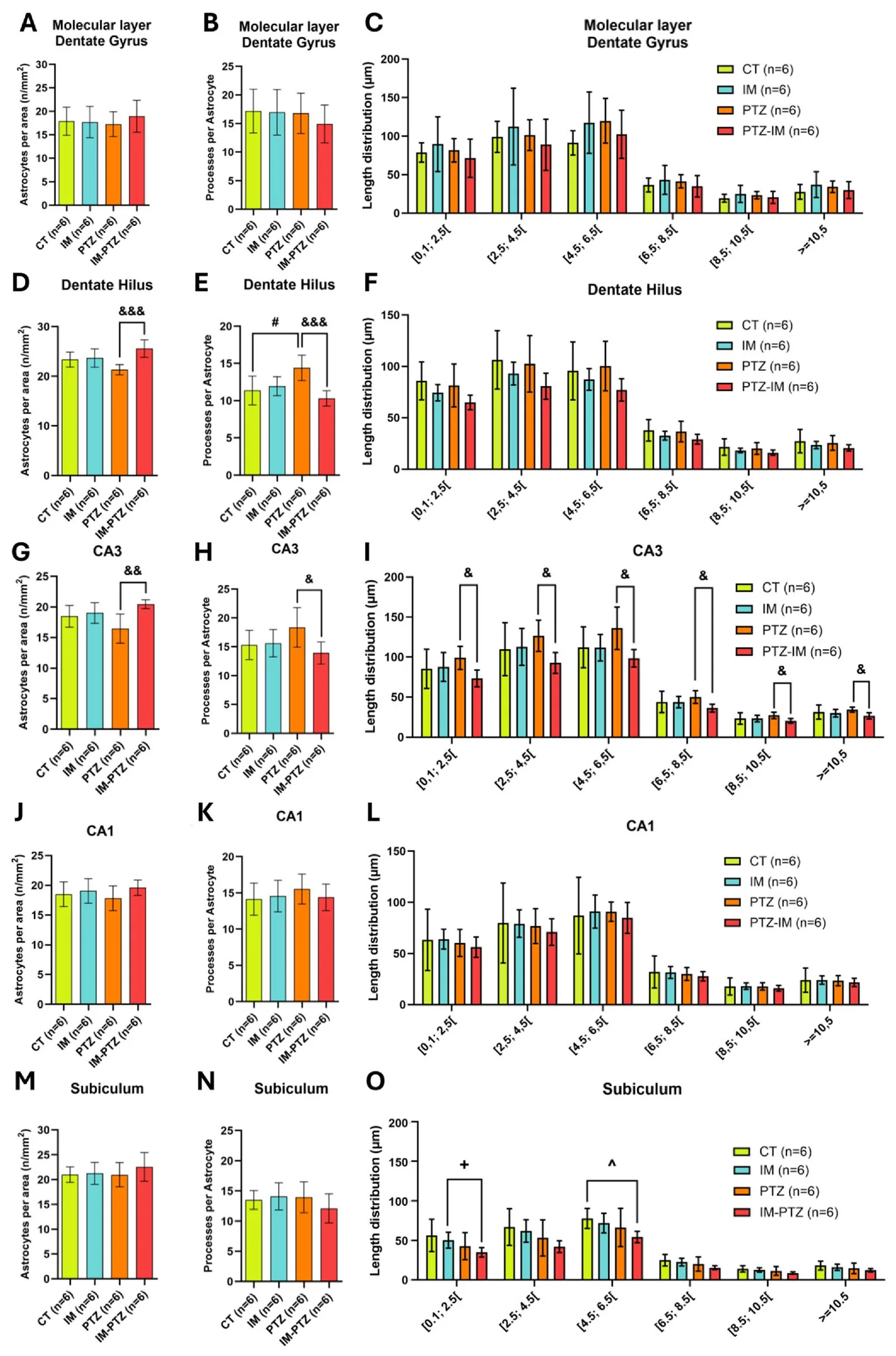
Results from astrogliosis evaluation. (**A**,**D**,**G**,**J**,**M**)—Histograms show astrocyte density in the different regions of the hippocampal formation (mean ± SD). Rats from the pentylenetetrazol (PTZ) group had a tendency towards lower astrocyte density compared to controls (CT) and imipenem-treated (IM) rats, albeit not significant. Imipenem after kindling (IM-PTZ) induced an increase in astrocyte density compared to PTZ-treated animals that was significant in the dentate hilus (*p* < 0.001) and CA3 (*p* < 0.01) regions. (**B**,**E**,**H**,**K**,**N**)—Histograms show number of processes per astrocyte in the different regions of the hippocampal formation (mean ± SD). Rats from the PTZ group had a significantly higher number of processes per astrocyte compared to the CT group in the dentate hilus (*p* < 0.05). IM-PTZ treatment induced a reduction in the number of processes per astrocyte compared to PTZ treatment that was significant in the dentate hilus (*p* < 0.001) and CA3 (*p* < 0.05) regions. (**C**,**F**,**I**,**L**,**O**)—Histograms show process length distribution in the different regions of the hippocampal formation (mean ± SD). In the CA3 region, IM-PTZ animals showed reduced process lengths than PTZ animals across both short/intermediate and long intervals (*p* < 0.05). In the subiculum, IM-PTZ animals also exhibited reduced values compared to IM and CT animals in specific short and intermediate intervals (*p* < 0.05), respectively. ^^^ *p* < 0.05 IM-PTZ vs. CT; ^+^ *p* < 0.05 IM-PTZ vs. IM; ^#^ *p* < 0.05 PTZ vs. CT; ^&^ *p* < 0.05, ^&&^ *p* < 0.01, ^&&&^ *p* < 0.001 IM-PTZ vs. PTZ.

**Figure 5:**
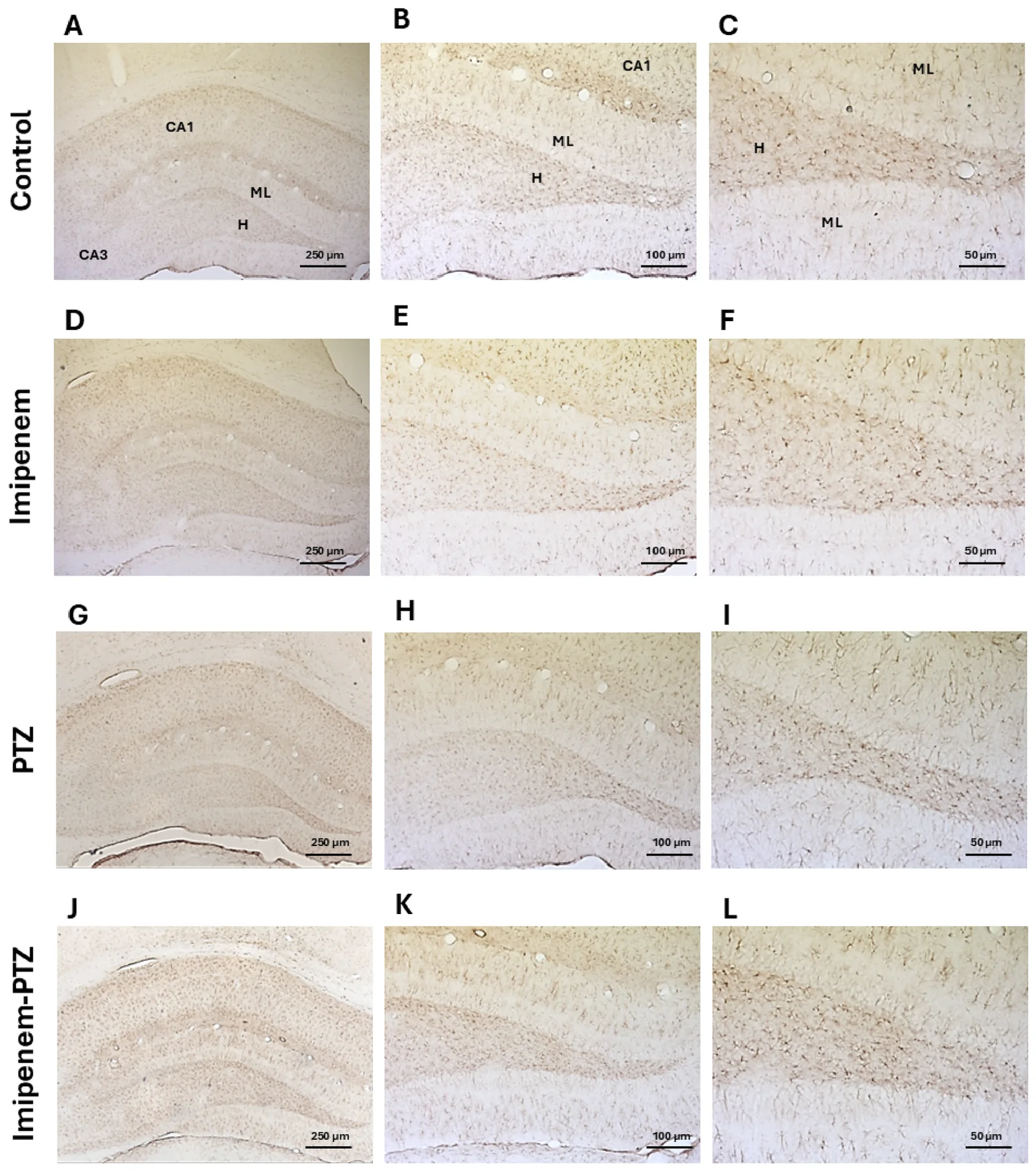
Astrogliosis evaluation in the hippocampus. Representative photomicrographs of the hippocampus from the different experimental groups at different magnifications. Labels are included only in the first photomicrographs for reference and are omitted from the remaining images for clarity. (**A**–**C**) CT group, (**D**–**F**) IM group, (**G**–**I**) PTZ group, and (**J**–**L**) IM–PTZ group. ML, molecular layer of the dentate gyrus; H, dentate hilus; CA3, pyramidal layer of the CA3 hippocampal field; CA1, pyramidal layer of the CA1 hippocampal field.

To assess whether the administration of PTZ and imipenem, alone or in combination, can elicit astrocytic activation or neuroinflammation, astrocytes’ areal density was quantified across the dentate gyrus, CA3, CA1 and subiculum (Figure 4A,D,G,J,M and Figure 5). One-way ANOVA revealed a significant effect of drug administration on the dentate hilus (F(3, 20) = 7.261, *p* < 0.01) and CA3 region (F(3, 20) = 5.367, *p* < 0.01), but not on the molecular layer of dentate gyrus (F(3, 22) = 0.3607, *p* = 7820), CA1 region (F(3, 20) = 0.9561) and subiculum (F(3, 22) = 0.7017, *p* = 0.5611). Albeit not significant, PTZ induced a trend towards reduction in the areal density of astrocytes in the dentate hilus, CA3 and CA1 regions compared to controls and imipenem-treated rats. More important, imipenem treatment of PTZ rats significantly increased the astrocyte areal density in the dentate hilus (*p* < 0.001) and CA3 (*p* < 0.01) regions compared to PTZ rats. In the remaining regions, this tendency towards an increase did not reach statistically significant levels.

Astrocyte morphology was characterized by assessing the number of processes per astrocyte (Figure 4B,E,H,K,N). One-way ANOVA revealed that there were significant effects of drug administration on the dentate hilus (F(3, 20) = 7.749, *p* < 0.01) and CA3 (F(3, 20) = 3.356, *p* < 0.05) regions, but not in the molecular layer of dentate gyrus (F(3, 22) = 0.5445, *p* = 0.6570), CA1 (F(3, 20) = 0.5165, *p* = 0.6756), and subiculum (F(3, 22) = 1.116, *p* = 0.3640). PTZ induced a significant increase in the number of processes per astrocyte in the dentate hilus when compared to controls (*p* < 0.05), as revealed by post hoc analyses. Moreover, imipenem administration in PTZ animals induced a t reduction in the number of processes per astrocyte in generally all the regions of the hippocampus with the exception of CA1, which achieved a significant level in the dentate hilus (*p* < 0.001) and CA3 (*p* < 0.05) regions when compared to PTZ-treated rats.

We further studied astrocyte morphology by analysing the length distribution of astrocytic processes, as shown in Figure 4C,F,I,L,O. Two-way ANOVA revealed a significant effect on process length in all hippocampal regions, including the granular layer of the dentate gyrus (F(1.219, 26.83) = 291.8, *p* < 0.0001), dentate hilus (F(1.348, 29.65) = 525.7, *p* < 0.0001), CA3 (F(1.640, 36.07) = 526.1, *p* < 0.0001), CA1 (F(1.224, 26.94) = 350.0, *p* < 0.0001), and subiculum (F(1.676, 35.20) = 275.7, *p* < 0.0001). A significant main effect of drug administration was observed in the CA3 region (F(3, 22) = 3.53, *p* < 0.05), but not in the granular layer of the dentate gyrus (F(3, 22) = 0.6514, *p* = 0.5905), dentate hilus (F(3, 22) = 1.868, *p* = 0.1645), CA1 (F(3, 22) = 0.1892, *p* = 0.9026), or subiculum (F(3, 21) = 2.583, *p* = 0.0805). Similarly, a significant interaction between drug administration and process length was detected in the CA3 region (F(4.919, 36.07) = 2.36, *p* < 0.05), but not in the granular layer of the dentate gyrus (F(3.658, 26.83) = 1.431, *p* = 0.2526), dentate hilus (F(4.043, 29.65) = 2.035, *p* = 0.1144), CA1 (F(3.673, 26.94) = 0.2640, *p* = 0.8853), or subiculum (F(5.028, 35.20) = 2.034, *p* = 0.0973). Post hoc analysis revealed that, in the CA3 region, IM-PTZ animals exhibited significantly lower values across multiple process length intervals compared to PTZ animals (*p* < 0.05), including both short/intermediate (0.1–10.5 µm) and longer processes (>10.5 µm; *p* < 0.01). Additionally, in the subiculum, IM-PTZ animals showed significantly lower values than IM animals in the shortest process length interval ([0.1–2.5] µm, *p* < 0.05). Rats from the IM-PTZ group also exhibited lower values than CT animals in the intermediate interval ([4.5–6.5] µm, *p* < 0.05). No significant differences were observed in the remaining length intervals.

## 3. Discussion

The present study demonstrates that imipenem can modulate hippocampal plasticity in a PTZ-kindled brain, interfering at the cellular level with both the neurogenic response and astrocytic architecture. While imipenem alone did not induce marked behavioural changes, it seems that its administration in the chronic epileptic model resulted in cognitive impairments in spatial learning and working memory that were predominantly driven by the underlying PTZ substrate, rather than a synergistic functional decline. Moreover, at the morphological level, imipenem suppressed the tendency towards a compensatory increase in hippocampal neurogenesis (evaluated through the density of DCX-positive neurons) typically triggered by PTZ, bringing values to baseline. Furthermore, in the dentate hilus and CA3 subfields, the combination of imipenem and PTZ induced a distinct “uncoupling” of the astrocytic response, characterized by increased cell density but diminished process complexity, instead of a healthy tissue normalization. These findings suggest that imipenem modulates cellular plasticity and microenvironmental homeostasis when administered upon a pre-existing pro-epileptogenic substrate.

### 3.1. Cognitive and Anxiety Effects of Imipenem in PTZ-Treated Rats

Notably our results showed that imipenem induced significant impairments in spatial learning and working memory only in rats previously sensitized with PTZ, indicating that its cognitive effects may depend on a pre-existing state of neuronal susceptibility. These results suggest that imipenem may impair cognitive performance primarily in conditions where neural networks are already sensitized or functionally compromised. Conversely, imipenem-treated rats that did not receive PTZ did not exhibit cognitive deficits.

In the present study, PTZ-kindling alone fails to induce significant impairment of MWM spatial learning and memory. This confirms prior studies where PTZ-kindled rats exhibited baseline performance similar to controls on the reference memory learning task [29]. It is important to note that there are also studies that showed that PTZ-kindling by itself could induce impairments in spatial learning and memory [30]. This divergence in PTZ-induced effects may stem from different protocols, animal age, or even variation in the efficacy of PTZ-kindling itself [31]. At the cellular level, PTZ drives neuronal excitability within the CNS by blocking GABAergic inhibition [26]. Single-dose administration of PTZ is sufficient to trigger status epilepticus but rarely precipitates chronic neurological alterations [32]. Conversely, chronic alterations and lasting susceptibility are reliably achieved through repeated, low-dose PTZ regimens [32]. Indeed, performance alterations in the MWM caused by PTZ are related not only to doses but also duration of treatment and the rat’s age [33]. However, although it is not significant, it is possible to observe a tendency towards a worse performance by PTZ-treated rats in the spatial learning task probe trial. Importantly, subsequent imipenem administration in PTZ-kindled rats appears to exacerbate PTZ-induced hyperexcitability. This synergistic effect resulted in significant spatial learning deficits, mirroring outcomes observed in models exposed to prolonged PTZ administration [34].

The analysis of the acquisition phase curves further elucidates the nature of this cognitive impairment. While all experimental groups showed a progressive reduction in latency in finding the platform—indicating that the basic ability to acquire the task was preserved—exploratory analysis of the final acquisition phase (trial blocks 6 and 7) revealed that the IM-PTZ group exhibited a significantly higher latency plateau. Although the global repeated-measures ANOVA was non-significant, the divergence observed in the final trial blocks suggests that the omnibus test may lack the sensitivity to capture localized deficits in spatial stabilization. These findings should be viewed as exploratory, raising the hypothesis that the combined pharmacological stress of PTZ and imipenem may specifically hinder the refinement of spatial maps and cognitive flexibility, rather than abolishing the memory of the platform’s location entirely. This is consistent with our probe trial results, where the lack of significant differences in platform crossings suggests that the fundamental spatial representation remains intact. Therefore, we hypothesize that the observed late-stage latency plateau may reflect a specific vulnerability in the optimization of navigation strategies, a hypothesis that warrants further investigation in future studies.

Relative to short-term memory, our results showed that PTZ-kindling induced significant impairment in working memory, corroborating previous studies [35], but contrasting with previous PTZ-kindling research that reported no significant differences [29]. These earlier experiments assessed short-term memory using Y-test, which is less sensitive to hippocampal impairment than the MWM working memory protocol [29,36]. While Y-maze assesses short-term memory based on spontaneous decision and exploration, the MWM focuses on active spatial navigation [29,37]. More importantly, in our study, PTZ-kindled animals treated with imipenem also showed a significant impairment on the working memory task, suggesting that the combination of PTZ-induced hyperexcitability and imipenem-related GABAergic interference may disrupt the rapid acquisition of spatial information. Spatial learning on the MWM depends strongly on hippocampal synaptic plasticity and memory consolidation across trials, which may be further disrupted by the combined effects of PTZ-induced hyperexcitability and imipenem-related GABAergic interference [37]. Interestingly, no significant differences were observed between the PTZ-alone and IM-PTZ groups in this task, with both displaying comparable working memory deficits. This lack of further exacerbation by imipenem suggests a potential ceiling effect, wherein PTZ-induced kindling alone severely disrupts short-term hippocampal plastic mechanisms, leaving a narrow margin for additional pharmacological impairment [38]. Alternatively, these results may indicate that imipenem exerts no deleterious effect on short-term spatial processing, demonstrating that the synergistic neurotoxicity of imipenem and PTZ is highly selective for long-term consolidation mechanisms (reference memory), whereas working memory remains predominantly vulnerable to the underlying pro-epileptogenic substrate alone.

Regarding anxiety, our findings suggest that the interaction between PTZ-kindling and imipenem may modulate the emotional profile. Performance in the open-field test showed comparable levels of the time spent in the both open-field zones across all groups, ruling out generalized open-field anxiety or gross locomotor deficits, which is consistent with previous works [39]. In the EPM, however, zone-specific significant differences were observed, with the PTZ group spending significantly more time in the closed arms compared to controls and imipenem groups. These results are consistent with previous works demonstrating the anxiogenic potential of the PTZ-kindling model [40,41]. Even though the IM-PTZ group showed a tendency to increase the time spent in the closed arms, this difference was not significant compared to control and imipenem groups. This suggests that imipenem subtly blunts the robust anxiogenic drive of PTZ, or that the emotional response in the combined group is partially masked by internal variability. The lack of behavioural changes in the open field test suggests that neither the seizures, imipenem nor the combination of both produces significant effects on gross locomotor activity, confirming that the observed deficits are specifically cognitive and emotional and not a consequence of impaired motor performance.

### 3.2. Effects of PTZ-Kindling and Imipenem on Neurogenesis in the Dentate Gyrus

Evaluation of neurogenesis through the density of DCX-IR neurons revealed crucial alterations in the hippocampal neurogenic niche. Our results show a tendency towards an increase in DCX-IR neuronal density in PTZ-kindled rats compared to controls and imipenem groups. A significant difference was only found between PTZ-kindled rats. Subsequent treatment with imipenem decreased DCX-IR neuronal density values comparable to baseline in the IM-PTZ group. DCX is a microtubule-associated protein transiently expressed in precursor and migrating immature neurons in the dentate gyrus, making it a reliable marker of adult neurogenesis [42,43]. Intact neurogenesis is vital for specific aspects of hippocampal function, including pattern separation and memory consolidation, and its disruption can compromise performance in spatial navigation tasks [44,45].

Mild seizures or early-stage PTZ-kindling often triggers compensatory reactive neurogenesis in the hippocampus, while chronic seizures can cause more severe aberrant alterations or even decreased neurogenesis, as the brain attempts to counteract excitotoxic stress [46]. Although statistically non-significant, the upward trend in DCX expression observed in our PTZ-alone group aligns with previous studies reporting an early upregulation followed by the normalization of neurogenesis during kindling, coinciding with the progressive depletion of pluripotent neurogenic cells [47,48]. The absence of significant alterations caused by imipenem in naïve rats is also congruent with previous experiments, where imipenem did not cause alterations in DCX-IR neuronal density [49]. Crucially, the significant drop in DCX-IR density in the IM-PTZ group compared to the PTZ-alone group suggests that imipenem interferes with adaptive neurogenic plasticity, thereby preventing the hippocampus from maintaining its proliferative response and bringing DCX levels back toward baseline.

Interestingly, this significant histological difference between the two kindled groups did not translate into a widespread behavioural divergence, as both IM-PTZ and PTZ-alone rats displayed remarkably similar profiles in the working memory task and the probe trial, with the combined group only underperforming controls during the final trial blocks of the acquisition phase. This close alignment suggests that while imipenem actively interferes with the neurogenic response triggered by PTZ, its additional impact on functional behaviour remains limited. Therefore, it seems that the observed cognitive impairments appear to be predominantly driven by the underlying PTZ-induced epileptogenic substrate, with imipenem acting as a subtle modulator of cellular plasticity rather than an aggressive driver of further behavioural deterioration.

The reduction in DCX-IR neuronal density in the combined PTZ and imipenem group compared to PTZ, in association with the latent network alterations, highlights the complexity of these overlapping treatments. Most of the adverse effects of imipenem are associated primarily with GABA receptor antagonism [50], a mechanism also shared by PTZ [51]. Optimal functioning of hippocampal networks relies on regulated inhibitory activity by the GABAergic interneurons; hence, the synergistic antagonism of GABA is likely one of the mechanisms underlying the memory impairments we found [52]. Network hyperexcitability might impair neuronal activity synchronization and reduce synaptic plasticity, thereby hindering long-term potentiation, which is key for learning and memory [53,54]. Consequently, impaired learning can be related to unregulated excitation, increased oxidative stress, and a breakdown of neurogenic compensation [55].

### 3.3. Astrocyte Response to PTZ-Kindling and Imipenem

GFAP is a primary cytoskeletal protein expressed in astrocytes and is commonly upregulated in conditions of neurotoxicity, including those resulting from brain injury or neurological disease [56]. Results from our experiment indicate that the astrocytic response to chronic hyperexcitability, caused by PTZ, is significantly modulated by the administration of imipenem. Although imipenem alone did not significantly affect astrocyte parameters, its interaction with PTZ-kindling was marked by distinct morphological and density-related changes.

Previous studies have shown that kindling with PTZ may cause no significant widespread astrogliosis nor massive increase in the expression of GFAP [57,58]. However, astrogliosis is a complex process involving not only the number of astrocytes but also their morphology, namely the number of processes [59]. In our study, PTZ-kindling alone led to a significant decrease in astrocyte density in the dentate hilus and CA3, which was accompanied by a significant increase in the number of processes per cell when compared to controls and IM-PTZ in the hilus, and IM-PTZ alone in CA3. This suggests that, during the PTZ-kindling, the brain attempts to maintain homeostatic support through increased branching despite a lower cell density.

Conversely, the administration of imipenem to PTZ-kindled rats altered this cellular profile. In the IM-PTZ group, we observed a return of astrocyte cellular density to values comparable to control and IM groups. Furthermore, the number of processes, however, was significantly reduced in the IM-PTZ group when compared directly to the PTZ-alone group in both regions but once again comparable to baseline values. Further analysis focusing on the length distribution of astrocyte processes showed significant differences in CA3 and subiculum. In CA3 the length distribution of processes from the PTZ group was uniform, with significantly more processes when compared to IM-PTZ. Nonetheless, neither the PTZ nor IM-PTZ groups differed from control and IM groups. In the subiculum, however, the distribution was uneven, as processes of IM-PTZ rats were less abundant in some of the shorter segments when compared to control and IM groups. This “uncoupling” between increased astrocyte density (IM-PTZ vs. PTZ) and diminished process complexity in the dentate hilus and CA3 is a known feature of maladaptive reactive astrogliosis [60], and such morphological shifts have been previously documented in several neurological diseases [61]. Much like the neurogenic profile discussed earlier, this distinctive cellular remodelling in the IM-PTZ group did not translate into an ongoing aggravation of cognitive decline when compared directly to the PTZ-alone group. Given that both kindled groups displayed remarkably similar performances in the working memory task and the probe trial—with the combined group only underperforming controls during the late acquisition blocks—it appears the significant morphological changes in astrocytes represent a cellular-level modulation rather than a strong structural driver of further functional impairment. Therefore, core behavioural deficits remain predominantly dictated by the underlying PTZ-induced epileptogenic substrate, whereas imipenem acts as a subtle modulator of the underlying glial response. Our analysis of CA1 and the molecular layer of the dentate gyrus revealed no significant changes in either astrocyte density or the number of processes, suggesting that the synergistic effects of PTZ and imipenem are regionally selective within the hippocampal formation.

Astrocyte activation is associated with increased neuroinflammation and is well documented in autoimmune [62], traumatic [63] and degenerative neurological diseases [64], as well as in epilepsy [65]. Increased hippocampal neuroinflammation is a reactive phenomenon to CNS insults, and is characterized by increased pro-inflammatory cytokines, impairment of normal synaptic plasticity and long-term potentiation, which are key to memory formation [66]. Given the proconvulsant properties of imipenem, the baseline-like astrocyte density and normalized process count in IM-PTZ rats suggest that the neuroinflammatory cascade typically triggered by PTZ (a known pro-inflammatory agent) may have been disrupted [59,67].

While severe astrocytosis and associated neuroinflammation clearly drive behavioural deficits, low-grade astrocytosis and neuroinflammation are rarely associated with robust alterations in anxiety models [68,69,70]. As our results suggest, PTZ-kindling in association with imipenem may dysregulate the normal neuroinflammatory responses and might also be a contributor to the cognitive impairments in memory observed in our study. Moreover, the documented correlation between an active astrocyte response and impaired MWM performance further supports our hypothesis [71].

### 3.4. Synthesis

In conclusion, our study provides evidence that lower, yet relevant, doses of imipenem can modulate hippocampal plasticity in a pre-sensitized brain, without inducing a widespread exacerbation of functional cognitive deficits. The synergy between PTZ-induced susceptibility and imipenem leads to an interference in neurogenesis and causes an uncoupling in the astrocyte response, returning cellular metrics to baseline values but compromising adaptative plasticity. Considering current knowledge, these findings can be explained by interference in the GABAergic system by both PTZ alone and PTZ in association with imipenem. Blocked GABAergic inhibition impairs the normal inhibitory regulation of hippocampal neuronal circuits, disrupting excitatory–inhibitory balance and promoting a dysregulated glutamatergic excitatory state [26,27]. These findings underscore the importance of careful antibiotic selection in patients with pre-existing neurological conditions or lowered seizure thresholds, as the impact extends beyond acute seizures to include subtle alterations in neurogenic, neuroinflammatory, and cellular homeostasis that may sustain underlying cognitive vulnerabilities.

## 4. Materials and Methods

### 4.1. Animals and Housing

A total of 52 five-month-old male Wistar rats were obtained from the Institute for Molecular and Cell Biology (Porto, Portugal). Rats were housed under standard controlled environmental conditions, a temperature of 23 ± 1 °C, relative humidity of 45 ± 5%, and a 12 h light/12 h dark cycle (lights on at 07:00 h and off at 19:00 h). Standard laboratory chow and water were provided ad libitum throughout the study. Rats were distributed among the four groups, with the PTZ-treated groups containing a higher number of animals to account for potential mortality during treatment that could otherwise compromise the experiments. The PTZ-treated groups had 14 rats each vs. the PTZ-naïve groups, which had 12 rats.

### 4.2. Drug Preparation and Administration

The PTZ was dissolved in sterile 0.9% sodium chloride (NaCl) solution at a concentration adjusted individually to ensure a maximum injection volume of 2 mL per rat. PTZ was administered by intraperitoneal (i.p.) injection using 25-gauge needles. This dose was based on previously used kindling protocols for the study of epilepsy and its treatment [25].

Diazepam was used as an abortive anticonvulsant therapy at a dose of 3 mg/kg given rectally.

Imipenem was administered at a dose of 40 mg/kg, prepared at a concentration of 10 mg/mL. The dose of 40 mg/kg of imipenem was selected based on its established neurotoxic profile in rodent models, as previously described by Golchin et al. [20]. Rats from both imipenem groups received one daily i.p. injection for 10 consecutive days. To avoid potential confounding factors such as physiological stress or neuroinflammation associated with invasive sampling—which could interfere with the subsequent behavioural assessments—no direct pharmacokinetic measurements (plasma or CSF) were performed in this cohort [72,73].

### 4.3. PTZ-Kindling Protocol

A PTZ-induced kindling protocol was used to induce progressive epileptogenesis through repeated subconvulsive stimulation. Rats received a total of up to 15 subconvulsive PTZ injections (35 mg/kg, i.p.) administered on alternate days. The selected dose (35 mg/kg) was intended to avoid generalized tonic–clonic seizures during the initial administrations but enough to provoke pro-epileptogenic alterations in most animals. Following each PTZ injection, animals were individually observed for 30 min to assess seizure activity. On day 31 (i.e., after the kindling period), animals received a single convulsive PTZ dose of 60 mg/kg (i.p.) to assess acquisition of the kindled state. Severity was evaluated using the Racine scale [74].

Kindling was considered established if rats met one of the following criteria: reaching Racine stage 4 or higher in three consecutive PTZ administrations, spontaneous seizures without PTZ administrations, or generalized seizures following the final convulsive validation dose. Based on previous literature, some animals may reach the kindled state before completion of the 15 subconvulsive injections [75]. In such cases, once kindling criteria were achieved, further subconvulsive injections were discontinued, and only the final validation dose was administered.

To prevent progression to status epilepticus (SE) and reduce mortality, diazepam (3 mg/kg) was administered rectally as abortive therapy when animals reached Racine stage 4 seizures. If seizure activity did not cease within 30–60 s after the first administration, an additional dose of diazepam (3 mg/kg) was administered. The objective was to prevent sustained SE and associated mortality while allowing seizure development sufficient for kindling induction.

### 4.4. Behavioural Testing

Following the treatment period, the rats underwent a series of behavioural assessments. The tests were conducted in the following sequence, with a one-day interval between each: MWM, working memory, open-field, and EPM. Prior to behavioural evaluation, all animals were handled daily for five consecutive days to facilitate habituation. On each testing day, rats were allowed to acclimate to the testing environment for a minimum of 30 min before the procedures began. All assessments were carried out during the light phase, beginning at 12:00 h, by two investigators who were blinded to the treatment allocation.

#### 4.4.1. Morris Water Maze (MWM)

##### Spatial Reference Memory Task

To assess spatial learning and memory, rats were tested using a maze consisting of a black circular pool (180 cm diameter; 50 cm deep) filled with water at room temperature (21 ± 1 °C), virtually divided into four equal-sized quadrants and located in a corner of a room containing extra-maze cues. A black escape platform (10 cm in diameter) was submerged, 2 cm below the water surface, in one of the four quadrants. Swim paths were recorded by a computerized video-tracking system (EthoVision XT 8.5, Noldus, Wageningen, The Netherlands).

During the place learning phase, rats were trained to locate a hidden escape platform positioned beneath the water’s surface. Animals completed one training session per day over 14 consecutive days. At the start of each trial, each rat was introduced into the pool facing the wall from one of four predetermined starting locations, presented in a pseudorandom sequence so that every starting point was used once within each set of four trials. Rats were given up to 60 s to locate the submerged platform. If unsuccessful within this time, they were gently guided to the platform by the experimenter and allowed to remain there for 15 s. Between daily trials, animals were placed in a clean holding cage for 30 s. Throughout the acquisition period, the platform remained in a fixed location. Swim path length was recorded for each trial as a measure of learning performance.

Twenty-four hours after the final acquisition session, spatial memory retention was assessed using a 60 s probe trial in which the escape platform was removed from the pool. Memory performance was evaluated by recording the number of crossings over the former platform location and the time spent in the target quadrant. On the following day, a visible platform test was conducted to assess sensorimotor function and visual performance. During this task, rats completed a single block of four trials separated by 30 s intervals. The platform, painted white and positioned 3 cm above the water surface, was relocated before each trial. The distance travelled to reach the visible platform was measured in each trial, and the mean value across all four trials was used for analysis.

##### Spatial Working Memory

Spatial working memory was evaluated using the delayed matching-to-place version of the Morris Water Maze, beginning three days after completion of the reference memory assessment. Animals underwent two trials per day for six consecutive days. In the first trial (information trial), the hidden platform was relocated each day to a new position, differing from the previous day’s location in both quadrant and distance from the pool wall. At the beginning of each trial, rats were released into the water facing the wall from the point farthest from the platform. Animals were given up to 60 s to locate the hidden platform. Those that failed to do so within the allotted time were gently guided to the platform by the experimenter and allowed to remain there for 15 s.

The second trial (retention trial) was conducted with the platform maintained in the same position as during the corresponding information trial. Rats again started from the location most distant from the platform, and all other testing procedures were identical to those used during the reference memory task. A 1 min interval separated the information and retention trials to assess short-term spatial memory retention.

#### 4.4.2. Open-Field Test

General locomotor activity and anxiety-related behaviour were evaluated using the open-field test. The apparatus consisted of a white acrylic arena measuring 100 × 100 × 40 cm. At the beginning of each session, each rat was placed in one corner of the arena and allowed to explore freely for 5 min. Locomotor activity was quantified by measuring the distance travelled in both the peripheral zone, defined as the area within 20 cm of the walls, and the central zone, corresponding to the 60 × 60 cm area located at the centre of the arena. Animal movements were recorded and analysed using the EthoVision XT 8.5 computerized video-tracking system (Noldus, The Netherlands). To eliminate olfactory cues, the apparatus was thoroughly cleaned and dried after each testing session.

#### 4.4.3. Elevated Plus-Maze (EPM)

Anxiety-like and exploratory behaviours were further assessed using the EPM. The apparatus was constructed from black acrylic and consisted of two opposing open arms and two opposing enclosed arms (50 × 12 cm) extending from a central platform measuring 12 × 12 cm. The enclosed arms were bordered by walls 50 cm in height. At the start of each trial, the rat was placed on the central platform facing a closed arm and was allowed to explore the maze freely for 5 min. Behavioural activity was recorded and analysed using the EthoVision XT 8.5 video-tracking system (Noldus, The Netherlands). The percentage of time spent and the distance travelled within the open arms, closed arms, and central platform were quantified. To prevent olfactory influences between animals, the maze was thoroughly cleaned and dried after every testing session.

### 4.5. Tissue Collection and Immunocytochemistry

Following completion of the behavioural assessments, approximately half of the animals in each experimental group were randomly selected for tissue collection (7 rats from each PTZ-treated group and 6 rats from each PTZ-naïve group). Animals were deeply anesthetized with sevoflurane (SevoFlo, Abbott Laboratories Ltd., Maidenhead, UK) before undergoing transcardial perfusion with 150 mL of 0.1 M phosphate buffer, followed by perfusion with 4% paraformaldehyde in phosphate buffer (pH 7.6) for tissue fixation. The brains were then carefully removed, coded to maintain blinding during subsequent processing and analysis, and divided into right and left hemispheres along the midsagittal plane. The anterior and posterior extremities were discarded, and the remaining tissue containing the hippocampal formation was preserved for subsequent immunohistochemical evaluation.

Taking into consideration previously described hemispheric asymmetries in the rodent hippocampus, tissue blocks were alternately obtained from the right and left hemispheres [76]. These hippocampal blocks were post-fixed for one hour in the same fixative used during perfusion and then cryoprotected overnight in a 10% sucrose solution at 4 °C. The tissue was subsequently mounted on a vibratome and sectioned coronally into serial slices 40 µm thick, which were collected in phosphate-buffered saline (PBS).

For each brain, two series of hippocampal sections were selected using systematic random sampling. The first section was randomly chosen among the initial 12 sections collected, and subsequent sections were obtained at consistent intervals of 480 µm (i.e., every 12th section) along the septotemporal axis of the hippocampus.

The remaining animals were also deeply anesthetized with sevoflurane and euthanized by decapitation. Immediately afterward, blood samples were collected by allowing blood to flow freely from the neck vessels into the collection tubes. In the case of perfuse animals, blood samples were obtained via cardiac puncture immediately before starting the process. These samples were centrifuged at 3000 rpm for 10 min at 4 °C, and the plasma fraction was separated, aliquoted, and stored at −80 °C for later biochemical analyses.

Immunohistochemical staining was carried out to evaluate the expression of DCX and GFAP. Free-floating brain sections were initially washed twice with phosphate-buffered saline (PBS) and incubated for 10 min in 3% hydrogen peroxide to suppress endogenous peroxidase activity. The sections were then incubated overnight at 4 °C with the respective primary antibodies: anti-DCX (Santa Cruz Biotechnology, Dallas, TX, USA cat. no. sc-271390; 1:500) or anti-GFAP (Dako, Carpinteria, CA, USA, cat. no. Z0334; 1:2000), both diluted in PBS containing Triton X-100.

After primary antibody incubation, the sections were rinsed with PBS and incubated with the corresponding biotinylated secondary antibody (Vector Laboratories; 1:400). This was followed by incubation with an avidin–biotin–peroxidase complex (Vectastain Elite ABC Kit, Vector Laboratories; 1:800). Both incubation steps were performed for at least 1 h at room temperature. Immunoreactivity was visualized using a chromogenic solution composed of 0.05% diaminobenzidine (DAB; Sigma) and 0.01% hydrogen peroxide. Between all incubation steps, the sections were washed in PBS for a minimum of 15 min. To facilitate antibody penetration, all incubation and washing solutions contained 0.5% Triton X-100.

All immunohistochemical procedures were performed simultaneously in 12-well culture plates, with four sections placed in each well, ensuring uniform processing conditions for all experimental groups. After completion of the staining procedure, sections were mounted onto gelatine-coated slides, allowed to air-dry, and dehydrated through a graded ethanol series (50%, 70%, 90%, and 100%). Finally, the slides were coverslipped using Histomount mounting medium (National Diagnostics, Atlanta, GA, USA).

### 4.6. Quantification of Areal Density of Doublecortin-Immunoreactive (DCX-IR) Neurons

Areal density estimates were obtained through microscopic analysis and manual drawing using a light microscope (Axio Scope.A1; Carl Zeiss Microscopy GmbH, Göttingen, Germany) fitted with a camera lucida (Carl Zeiss Microscopy GmbH, Göttingen, Germany) at a final magnification of ×160. Cells were classified as immunoreactive when their perikarya exhibited intense staining. DCX-IR neurons were quantified exclusively within the subgranular layer of the dentate gyrus.

The subgranular layer was consistently identified at all levels along the septotemporal axis of the hippocampal formation using cytoarchitectonic landmarks and reference to a rat brain atlas. This region was defined as a narrow band of tissue approximately 30 µm thick located between the granule cell layer and the hilus [77,78].

Quantitative estimates were derived from an average of 12 immunostained sections per animal, sampled according to the procedure described above. The surface area of each layer was estimated from camera lucida drawings. The cross-sectional area of each layer was estimated using the point-counting stereological method. A transparent grid with regularly spaced points was superimposed onto the drawings, and the number of points falling within the boundaries of each layer was counted for stereological area estimation. Finally, the total number of labelled cells was divided by the corresponding laminar area to obtain areal density values, expressed as cells per mm^2^.

### 4.7. Quantification of Astrocyte Morphology

Previously immunostained brain sections were imaged at a final magnification of 20× using a Zeiss Scope A.1 light microscope (Carl Zeiss Microscopy GmbH, Göttingen, Germany) fitted with an AxioCam MRc5 digital camera (Carl Zeiss Microscopy GmbH, Göttingen, Germany). The dentate gyrus, CA3, CA1, and subiculum were identified throughout the septotemporal extent of the hippocampal formation based on their characteristic cytoarchitecture, with anatomical boundaries established according to a standard rat brain atlas [77,78]. For the purposes of analysis, astrocytes located in the CA2 region were included within the CA3 area.

Digital images were acquired using AxioVision Rel. 4.8 software (Carl Zeiss Microscopy GmbH, Göttingen, Germany) and subsequently analysed with ImageJ (version 2.16; National Institutes of Health, Bethesda, MD, USA). A custom-designed macro was employed to quantify GFAP-immunoreactive (GFAP-IR) astrocytes and to evaluate morphological characteristics, including astrocyte soma area and the length of their cellular processes [79].

### 4.8. Data Analysis

Behavioural results are expressed as mean ± standard error of the mean (SEM), whereas morphometric data are presented as mean ± standard deviation (SD). Statistical analyses and graphical representations were performed using GraphPad Prism 10.6.0 (GraphPad Software, La Jolla, CA, USA). A repeated-measures ANOVA was used to analyse Morris Water Maze performance. As an exploratory approach to characterize spatial memory consolidation, we also performed a one-way ANOVA followed by Tukey’s HSD post hoc test on the final acquisition trial blocks (6 and 7) to examine treatment-specific differences. A two-way ANOVA was applied to evaluate the effects of treatment and arena zone in the open-field and EPM tests. Data obtained from DCX-immunoreactive (DCX-IR) area density, as well as the number and morphological characteristics of GFAP-IR astrocytes, were analysed using one-way ANOVA with treatment as the independent factor. When significant main effects were detected, Tukey’s honestly significant difference (HSD) post hoc test was performed for multiple comparisons. Statistical significance was defined as *p* < 0.05 for all analyses.

## 5. Conclusions

This study demonstrates that the imipenem effects on cognition, neurogenesis and astrocyte response are dependent on the functional state of the brain. Imipenem in PTZ-kindled animals is associated with cognitive impairments in the MWM, whereas no significant cognitive alterations were found in healthy controls or animals exposed to imipenem alone. The results suggest that while the underlying PTZ-induced substrate is the primary driver of the observed memory deficits, the interaction between PTZ and imipenem specifically reshapes the underlying cellular landscape rather than provoking an aggressive, synergistic functional decline.

At a cellular level, imipenem effectively suppressed the adaptive tendency towards an increase in neurogenesis triggered by PTZ-kindling. This suggests that the associated cognitive deficits occur alongside a failure in maintaining neurogenic niche plasticity under pharmacological stress. Astrocyte analysis further revealed that imipenem modifies the glial response to PTZ-induced neuronal stress. While neither PTZ nor imipenem alone caused significant reactive changes, the combined treatment altered astrocyte number and morphology in the dentate hilus and CA3, inducing a distinct uncoupling between cell density and process complexity. These alterations may indicate changes in neuroinflammatory signalling or a compromised capacity of astrocytes to regulate synaptic homeostasis.

Taken together, these findings indicate that the detrimental neurobiological effects of imipenem emerge primarily in conditions of increased neuronal vulnerability, such as those associated with epileptogenic processes. The results highlight the importance of considering underlying neural susceptibility when evaluating the safety profile of β-lactam antibiotics. Future studies should further investigate the specific molecular mechanisms linking imipenem-induced GABAergic interference, astrocyte reactivity, and hippocampal network dysfunction, as well as explore potential neurogenic, neuroinflammatory, and oxidative pathways involved in these effects.

## Data Availability

The datasets generated and/or analysed during the current study are available from the corresponding author on reasonable request.

## Abbreviations

The following abbreviations are used in this manuscript:

ANOVA	Analysis of Variance
CA1	Cornus Ammonis 1
CA2	Cornus Ammonis 2
CA3	Cornus Ammonis 3
CNS	Central nervous System
DCX	Doublecortin
DCX-IR EPM	Doublecortin-immunoreactive Elevated Plus Maze
GABA	γ-Aminobutyric acid
GFAP	Glial fibrillary acidic protein
GFAP-IR	Glial fibrillary acidic protein-immunoreactive
IM	Imipenem-treated group
IM-PTZ	Imipenem-pentylenetetrazole-treated group
MWM	Morris water maze
PBS	Phosphate-buffered saline
PTZ	Pentylenetetrazole
SE	Status epilepticus
